# Frontiers Review: Severe Asthma in Adolescents

**DOI:** 10.3389/fped.2022.930196

**Published:** 2022-07-07

**Authors:** Sara Warraich, Samatha Sonnappa

**Affiliations:** Department of Respiratory Pediatrics, Royal Brompton Hospital, London, United Kingdom

**Keywords:** severe childhood asthma, severe adolescent asthma, holistic management, transition, collaborative working

## Abstract

Asthma remains the most prevalent chronic respiratory disease of childhood. Severe asthma accounts for a minority group of patients but with substantial morbidity burden. It may reflect disease which is resistant to treatment or that which is difficult to treat, or a combination of both. The adolescent patient cohort denote a unique group and are the focus of this review. This group of patients embody transitioning priorities and evolving health beliefs, all of which may influence the management and burden of disease. Factors of importance include the influence of physiological parameters such as sex and race, which have confer implications for medical management and non-physiological factors, such as adherence, risk-taking behavior, and vaping. The holistic approach to management of severe asthma within this group of patients must acknowledge the evolving patient independence and desire for autonomy and strive for a collaborative, patient tailored approach. This review will focus on the factors that may pose a challenge to the management of severe adolescent asthma whilst offering suggestions for changes in practice that might harness patient priorities and shared clinical decision-making.

## Introduction

Asthma is the most prevalent chronic lung disease of childhood (1). Most children with asthma achieve good symptom control with low-moderate dose inhaled corticosteroids (ICS). However, a small group with severe disease and poor control despite maximal treatment need further escalation in support and intervention.

The European Respiratory Society (ERS) /American Thoracic Society (ATS) defines *severe asthma* in those over 6 years of age, as that which obligates treatment with high dose ICS and another maintenance agent, or use of systemic steroids, for more than half of the previous year to achieve control (2).

Along with high treatment burden, severe asthma confers significant morbidity and mortality. Within pediatrics, the National Review of Asthma deaths in the UK revealed that mortality in this population remains alarmingly high (3). Asthma control may be rendered difficult due to a variety of factors that demand recognition (4, 5), particularly within the adolescent group (6).

## The Adolescent With Severe Asthma

The World Health Organization (WHO) defines *adolescence* to be within the ages of 10 and 19 years (7) whilst others propose a more extended length of 10–24 years a more accurate representation (8). Certainly, adolescence embodies young adulthood and a challenging time of transition, marked by the pursuit for independence (9) and autonomy with transformations in social, emotional, and physical domains (10). Ownership of health and self-care sharpens into focus, becoming a greater priority.

Marked differences can exist in the manifestation, exacerbating factors, and management strategies for asthma at different age groups (11). Furthermore, challenges in management lay bare the influence of disparities in socio-economic status, education, exposure to pollution and healthcare access, categories recognized by the Global Initiative for Asthma as obstacles to decreasing the disease burden (12). Ethnic differences in asthma outcomes have also been postulated, with implications for biologics and therapeutic possibilities (13). A study in pediatric minority populations revealed that higher IgE levels were significantly associated with severe asthma, poor control, and exacerbations in the Puerto Rican group whilst none of these outcomes were observed in Mexican Americans. Furthermore, eosinophilic asthma had links with greater asthma severity and exacerbations in the Puerto Rican group, inferring that eosinophil-targeting therapies may confer an advantage in this group (13). Additionally, influence of sex differences are documented, with notable reference to symptomology within the adolescent period (6). A shift in higher prevalence from males to females during the pubertal period is recognized, with greater occurrence of wheeze and more severe asthma in females (6). In an European Community Respiratory Health Survey study of adult female population, lower lung function and higher likelihood of asthma were observed in those with early menarche, implicating hormonal factors (14). Furthermore, asthma in pregnancy has been linked with obstetric complications (15), which may have significant implications for our female cohort of patients. Therefore, as our patients transition to young adults, the growing relevance of such findings should be considered.

This review will explore the multifaceted parameters that bear significance to the management of severe asthma in the adolescent group.

## Confirming The Diagnosis

### The Diagnostic Label

The WHO categorizes severe asthma into three groups; (1) inadequately treated (2) that which is difficult-to-treat and (3) severe treatment-resistant asthma (STRA), the last of which represents asthma for which the highest level of management is necessitated to achieve control, or which does not despite this (16).

A crucial initial step is in ensuring the diagnostic labeling is accurate and should begin with addressing the basics. A multidisciplinary approach is paramount (5) in diagnosing, initiating, and maintaining treatment.

STRA may warrant the use of biologics and in the UK currently omalizumab and mepolizumab are licensed for use in children aged above 6 years and dupilumab for children aged above 12 years, with the caveat that the patient has not shown an adequate response or is not eligible for mepolizumab (17). The US Food and Drug Administration (FDA) and European Medicines Agency (EMA) have licensed all three biologics for children above age 6 years. In addition, Benralizumab is licensed by the FDA and EMA for children aged 12 years and above with severe eosinophilic asthma. Difficult-to-treat asthma, however, necessitates a broader approach, one that embraces extra-pulmonary influences such as obesity, adherence, family values, and symptom perception (18). Moreover, several conditions might present an asthma-like façade and should addressed (5).

### Investigations

A thorough history and appropriate investigations facilitate diagnostic clarity. Investigations include blood tests for full blood count, total IgE and specific IgE to aeroallergens, as severe childhood asthma has associations with atopy and increased blood eosinophilia (19). Spirometry with bronchodilator reversibility is useful for diagnosis, although it is important to note that some children with asthma may have non-obstructive spirometry when not acutely unwell. Fractional exhaled nitric oxide (FeNO) is a surrogate measure of eosinophilic airway inflammation and a good predictor of corticosteroid response. Additional investigations such as inhaled methacholine challenge, eucapnic voluntary hyperventilation and cardiopulmonary exercise tests can provide further clarity. Flexible bronchoscopy may be indicated, to assess for anatomical variations including airway malacia and vascular rings (20). Analysis of the bronchoalveolar lavage (BAL) for cytology and culture growth is beneficial and children with STRA are noted to have a higher eosinophil count in the BAL compared to control subjects (21). Further exclusionary investigations including sweat chloride testing, immunoglobulin levels, testing for reflux, and video fluoroscopy for swallow assessment may be useful (20).

## Factors Of Influence In Adolescent Severe Asthma And Management

### Breathing Pattern Disorders

Several comorbidities may confound the presentation and diagnosis of asthma and should be considered. Of note are breathing pattern disorders. These are best described as chronic or recurrent changes in breathing pattern that cause respiratory symptoms such as breathlessness and non-respiratory symptoms such as anxiety, light-headedness, and fatigue. The prevalence of some breathing pattern disorders in adolescents with asthma may be as high as 25% (22). Disruption of an optimal breathing pattern can contribute to multiple distressing and debilitating symptoms that impact significantly on the quality of life. The symptoms can masquerade as asthma or worsen asthma symptoms particularly in those with exercise induced dyspnoea and during an acute asthma attack. Breathing pattern retraining can therefore be useful in the management of severe asthma.

### Obesity

Obesity, a global health concern, is a significant risk factor for severe asthma and is associated with poor asthma control and increased health care utilization (23). Systemic corticosteroid responsiveness is also impaired in obese children with asthma and is accompanied by heightened patterns of systemic inflammation and metabolites of oxidative stress (24). Additionally, obesity may be associated with other co-morbidities which may impact the holistic quality of life and influence management.

### Adherence

The aim of any asthma management strategy should be to achieve symptom control, with minimal therapeutic intervention. The WHO suggests adherence to be “the extent to which a person's behavior—taking medication, following a diet, and/ or executing lifestyle changes, corresponds with agreed recommendations from a health care provider” (25). Contrary to any paternalistic approach, a collaborative effort between the patient and professional, one which embraces the patient priorities, will likely underpin successful adherence. This is particularly relevant since this modifiable factor is recognized as a challenge among adolescents (26), contributing to uncontrolled disease. The National Review of Asthma Deaths in the UK highlighted that over half of asthma deaths were preventable through addressing avoidable factors, including non-adherence and missed appointments (3).

A compendium of factors sway adherence in adolescents (26) and in turn influence the management of severe asthma especially as they transition. Examples of such influences include the determination for greater independence and resistance to parental monitoring (26) paradoxically accompanied by forgetfulness and struggles with organization of time (26). This may also be augmented by social stigma and risk-taking behaviors.

Conflicting and changing priorities and attitudes to health play a key role in this age group (9, 26) and therefore, exploring and accommodating for factors that matter to adolescents may be critical in mitigating the burden of severe disease.

### Adherence Monitoring

Adherence to treatment may be viewed in stages; *initiation* (taking the first dose), *implementation* (the actual dosing by the patient in comparison to what is prescribed) and *persistence* (time from starting to eventually stopping treatment) (27). In the context of severe adolescent asthma, which may be subject to variable triggers and alterations to treatment regimens, these stages may be in a dynamic state, especially if compounded by non-adherence. Adherence is considered good if over 80% of the prescribed doses are taken with an appreciation that higher treatment observance is associated with reduced exacerbations (28). Adherence is acknowledged as lower in adolescents (26) and a study of 15–18 year olds with moderate to severe asthma, showed a median adherence of 43% detected through electronic monitoring (29). It is important to recognize that whilst non-adherence may be intentional, it may also be non-intentional (30), creating scope for a change in practice.

Furthermore, monitoring adherence may itself pose a challenge. A combination of history and investigations, including trends in spirometry and FeNO measurements, and prescription uptakes offer some indication. Subjective tools to ascertain treatment observance include self-reporting questionnaires, such as the Medicine's Adherence Report Scale (31). However, over reporting may be observed. Objective monitoring tools include data on prescriptions issued, weighing canisters and directly observed therapy (DOT) (31). Aside from DOT, the former measures do not, in fact, reveal the actual dosing of the treatment. Novel electronic monitoring devices (EMD) may enable clinicians to circumvent this challenge (32).

### Mental Health and Risk-Taking Behavior

Symptoms of depression and anxiety are documented in adolescents with asthma (33, 34) and are associated with lower quality of life (35). *Post-hoc* analysis of the IDEAL study noted that 81% of the patients with severe asthma had uncontrolled disease, which was associated with reduced lung function and worsened health related quality of life (35). Furthermore, greater acute healthcare utilization is also observed in adolescents suffering from depression (36, 37).

Worryingly, a higher percentage of risk taking behavior has been described in adolescents with severe asthma (26, 33) including smoking and substance misuse (33). A multi-state survey by the Centers for Disease Control and Prevention revealed that cigarette smoking, cocaine use and low mood were more prevalent in youths with asthma than without (38). Moreover, factors were recognized to co-influence, and where suicidal ideation was reported in those with asthma, there was an association with binge drinking and more than 60% of smoking marijuana (38).

These represent important potential confronts in managing adolescent severe asthma and an integrative approach, which interlocks different tiers of support including school and psychology, is imperative in providing holistic management.

### Symptom Perception

Finally, it is important to consider that whilst symptom severity has been described above, poor symptom perception and tolerance to high levels of symptoms is another challenge and may contribute to poor control (26). Poorer symptom control in adolescents has been observed, with the International Study of Asthma and Allergies in Childhood (ISAAC) noting a higher year-long prevalence for symptoms of exercise induced wheeze, severe wheeze limiting speech and night-time cough in adolescents, compared to younger children with asthma (39).

### A Growing Threat: E-Cigarettes and Electronic Nicotine Delivery Systems

The Unbiased Biomarkers for the Prediction of Respiratory Disease Outcomes (U-BIOPRED) study reiterate the deleterious impact of smoking on symptomology, noting the association of exhaled smoke with severe wheeze (40). However, vaping is another growing threat and E-cigarettes and Electronic Nicotine Delivery Systems (ENDS) are described as up to two-three times more popular amongst adolescents and young adults in comparison to older adults, despite the original ambition to support smoking cessation in adults (41). A study of young adults in school and colleges (42) revealed that the top promotive factors for e-cigarette experimentation included inquisitiveness, enticing flavors, and peer influences. Interestingly, the perception that these were a healthier alternative to traditional smoking promoted their use, whilst having health concerns was a deterrent (42). This highlights the unassailable influence of education on adolescent health behaviors, to facilitate a more informed transition into adult health care.

Whilst extensive research is lacking, the present understanding on vaping effects on the lungs highlights the associations with increased severe asthma attacks, airway inflammation, increased risk of acute lung injury and reduction in lung function (41). Two case descriptions of adolescents with severe asthma exacerbations resulting in veno-venous extracorporeal membrane oxygenation (VV-ECMO) with earlier ENDS exposure have been described (43), reinforcing the urgency in understanding the adverse effects.

Furthermore, E-cigarette use has been shown to be a *risk* factor for future smoking, not simply a *marker* for future smoking (44). Data on e-cigarettes from the Children's Health Study (45) reported that 40% of e-cigarette users commenced cigarette smoking, noting a 6 times higher odds of starting cigarettes smoking if they were e-cigarette users, compared to having never-used. More alarmingly, the relationship was stronger for those who had not planned to commence smoking prior to the e-cigarette use. Therefore, E-cigarettes and Electronic Nicotine Delivery Systems are a growing health threat for adolescents and holistic management should include this understanding.

## Discussion: Severe Adolescent Asthma Management—A Collaborative Approach

### Family Dynamic and Peers: Support Networks

Family dynamics and influence of peers is consistently recognized as an important factor. Stress at the family level is associated with adverse asthma outcomes (46) and parents of adolescents with severe asthma are recognized as more anxious than those without (47). The pursuit for a new equilibrium, in establishing new responsibility for medication adherence, may be subject to confusion in roles where the adolescent may reject the parental support but also rely on it (26). Parents can support the practicalities of management e.g., prescriptions (28) and are in a position to explain rationale and reasoning to the transitioning young adult (28), which would be beneficial.

Furthermore, peer interactions are a significant factor of influence and clinical management should seek to harness this stimulus positively. Emotions of embarrassment are recognized to influence adherence (28). More than half of non-adherent episodes were amongst friends in one study (48) and hesitation in participating in social activities has also been noted (28). Moreover, social media influences are a very relevant entity for the current adolescent cohort and the influence should be explored in clinical consultations.

### Motivational Interviewing and Co-design: Greater Ownership of Care

**Figure 1:**
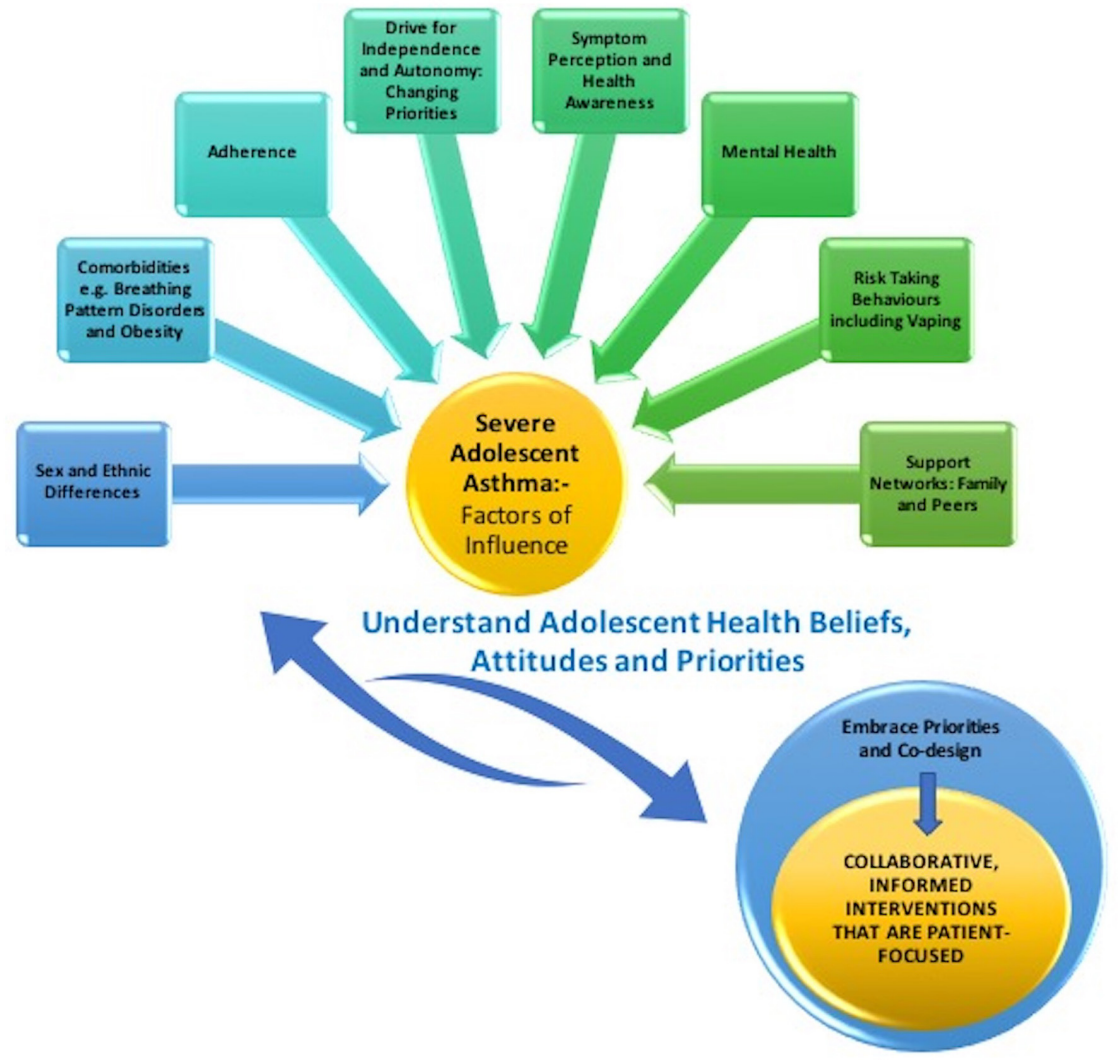
Illustration of the interplay between factors of influence for the adolescent patient with severe asthma and collaborative working.

Approach to severe adolescent asthma should embrace clinical necessity with adolescent priority, to cultivate relevant and sustainable interventions. Acknowledging that management does not rely simply on medical interventions but embraces the multifaceted influences discussed thus far in this review, offers key insight. Creating greater ownership of care should be a priority and listening to and engaging the adolescent patient should underpin all clinical consultations. Shared decision-making with patients has shown to enhance adherence amongst adults with poorly controlled asthma, as it accounts for their priorities (49). This is very relevant for our adolescent cohort, as they embrace greater autonomy in health care decisions. Figure 1 summarizes the proposed interplay between factors of influence and collaborative working with the adolescent patient.

The Health Belief Model (50) proposes that the actions of individuals balance their beliefs about the perceived health risk with the benefits of the behavioral changes required to address the threat. The susceptibility to the disease and the challenges to overcome in achieving the intended behavior change are important considerations and have been described in the context of asthma in adolescents (29). This study reviewed the perceptions around inhaled steroids and their impact on health, challenges to taking medications (e.g., taste, fear of addiction) and suggested prompts for progress (e.g., audio and visual reminders and incentives) (29).

Understanding adolescent health beliefs is therefore a crucial pedagogical step. Motivational interviewing (MI) may be employed, exploring individual priorities and promoting positive health behavior (30). It has been used with success in different settings amongst the adolescent cohort, including diabetes (51). Furthermore, when paired with social media, online support and other supportive adjuncts such as family contributions, MI outcomes are more effective (30).

Creating a bridge between patient held beliefs and accurate knowledge of disease and treatment is an important next step. Experiencing episodes of severe asthma exacerbations and decline in asthma control, has shown to emphasize the importance of maintenance therapy and motivate greater adherence in adolescents (28). This offers an example of health consequence and subsequent modification of behavior. In one study, despite regular attendances at medical appointments, some youths did not appreciate the rationale for medication use and whilst feeling involved, many did not take the ownership of their healthcare visit (52). This highlights an opportunity for timely engagement in self-care, mitigating learning through negative clinical experiences.

A further example where collaborative working may yield a more effective outcome may be seen in school attendance and symptom perception. School and educational establishments are likely to play an integral role in adolescent lives and educational accomplishments are recognized as a priority for adolescents (6). However, poor awareness of symptoms has a considerable impact on adolescent school attendance, with up to nine times greater chance of hospital admissions and four times greater likelihood of school absenteeism, in one study (53). Therefore, greater knowledge around symptom presentation and benefits of better control for the priorities that matter to adolescents, will enable shared clinical goals. This may also be harnessed within the practical components, such as inhaler technique, with poor technique shown to contribute to poor adherence and control (54).

Therefore, collaborative working with the adolescent patient and their families, one which endeavors to codesign clinically appropriate but patient-focused management regimens, may offer a bridge to more effective management of severe, difficult to manage asthma.

### Transition

Transition from pediatric to adult services is an important phase in the management of severe adolescent asthma and should endeavor to acknowledge the influence of the various aspects discussed in this review. The transition process should involve a named asthma nurse and consultant from the pediatric and adult services for better outcomes. Whilst a consensus on a set age for transition is lacking, it usually between the ages of 16 and 18. However, the conversations around transition should start early (11, 55), aiming for a gradual process rather than an abrupt event and offering time for readiness. Transition to adult services may be a worrying time for parents and carers, especially when their child's asthma has been difficult to control and they need to be supported in letting their adolescent have control over their asthma before transitioning to adult services. The emphasis should be on self-management by the adolescent with caregiver engagement, whilst acknowledging vulnerabilities (11). Co-production of relevant and informed transition services with young people and their carers is strongly advocated (55) and exploring patients' beliefs and goals is recognized as vital in enabling smooth transition (56). These interactions offer opportunities to identify and address potential challenges and barriers. Therefore, transition should embrace the priorities of the adolescent and family and aim to work in collaboration with all groups, including adult services, to achieve successful transfer of priorities, goals, and care.

### Future Directions and Challenges

Despite greater understanding, asthma mortality remains high and there are associations that need further exploration. The authors recognize that one such area is the contribution of ethnic differences in asthma subtypes and outcomes, with recent findings suggesting the influence of race and ethnic differences in asthma outcomes and severity (13). These findings expose the need for further research into the role of racial and ethnic differences in severe asthma disease management.

### Summary

Severe asthma in the adolescent requires a broader perspective and demands recognition of the components that are relevant to this unique age group. Table 1 summarizes the key learning points. Adolescence marks a period of transition and is subject to a variety of influences. Escalation in support may be medical such as the use of biologic therapy but may also warrant a wider approach, which nurtures collaborative decision making with the adolescent cohort and strives for more patient-relevant management regimens.

**Table 1:** Summary of key learning points.

**Key learning points: severe adolescent asthma** • Ensure that the diagnosis of asthma is correct, with a multi-disciplinary approach to diagnosis and management. • Adolescence marks a transition toward greater independence and autonomy in decision making. • Factors of influence include family dynamics, peer influences, health beliefs and impact of comorbidities including breathing pattern disorders. • A collaborative approach, which combines adolescent priorities with stronger health education and treatment rationale, is integral to promoting successful ownership of care and adherence. • Holistic management must acknowledge the evolving trends of relevance for our adolescent cohort, including social media and vaping.

## Author Contributions

SW wrote the first draft. Both authors reviewed, revised, made contributions to all subsequent drafts, and contributed to the work of this manuscript.

## Conflict of Interest

The authors declare that the research was conducted in the absence of any commercial or financial relationships that could be construed as a potential conflict of interest.

## Publisher's Note

All claims expressed in this article are solely those of the authors and do not necessarily represent those of their affiliated organizations, or those of the publisher, the editors and the reviewers. Any product that may be evaluated in this article, or claim that may be made by its manufacturer, is not guaranteed or endorsed by the publisher.
